# Salting-Out Effect Behavior of Protein/λ-Carrageenan Composite Gels Enhanced by Enzymatic Pretreatment: Focusing on Microstructure, Interactions and the Potential for Dysphagia Food

**DOI:** 10.3390/foods14213662

**Published:** 2025-10-27

**Authors:** Bowen Yang, Shicheng Dai, Yaqi Tang, Tianhe Xu, Junzheng Wang, Weixiang Zhu, Junfeng Xie, Xiaohong Tong, Huan Wang, Lianzhou Jiang

**Affiliations:** College of Food Science, Northeast Agricultural University, Harbin 150030, China; 13946087819@163.com (B.Y.); 18443602143@163.com (S.D.); a15009803347@163.com (Y.T.); 15830825739@163.com (T.X.); 18245323025@163.com (J.W.); 18246174056@163.com (W.Z.); 18845157820@163.com (J.X.); tongxiaohong0110@163.com (X.T.)

**Keywords:** soybean protein isolate, λ-carrageenan, enzymatic hydrolysis, salting-out, dysphagia gel

## Abstract

This study aimed to investigate the effects of synergistic K^+^ immersion-induced salting-out on the rheological properties, microstructure, molecular interactions, and swallowing adaptability of soy protein isolate (SPI)/λ-carrageenan composite gels under different enzymatic pretreatment times (0, 10, 20, 30, 60, and 120 min) using Flavourzyme. The results showed that enzymatic hydrolysis increased the degree of hydrolysis of SPI from 1.11% to 11.46%, gradually degraded the 7S subunit, and reached the highest surface hydrophobicity at 30 min of moderate hydrolysis. After KCl immersion treatment, the K-λ/SPI gels exhibited lower water holding capacity and higher whiteness compared to those before immersion. Among them, the K-λ/SPH30 group demonstrated the best rheological properties. Moderate enzymatic hydrolysis synergistically promoted the formation of a dense network in K-λ/SPI gels. This process enhanced the stability of the composite gel through hydrophobic interactions, electrostatic interactions, and hydrogen bonds while simultaneously increasing the proportion of β-structures (reaching a maximum of 62.05%). The expanded binding sites from moderate enzymatic hydrolysis, combined with the dense network and enhanced interactions, collectively strengthened the salting-out effect. This ultimately enabled K-λ/SPH30 to achieve the highest crystallinity (93.57%), the highest K^+^ content (4.80%), and the optimal swallowing performance (IDDSI level 5). This study not only deepens the understanding of the synergistic mechanism between protein hydrolysates and salting-out but also provides an innovative solution for designing foods for dysphagia diets.

## 1. Introduction

Soybean protein isolate (SPI) is a high-quality plant protein that occupies an irreplaceable position in the food industry due to its high nutritional value and low production costs. However, research found that SPI has a compact amorphous structure, and a large amount of protein in it is in an aggregated state, which directly limits the gel properties of SPI and requires appropriate modification methods [1,2]. Chemical, physical, and enzymatic treatment methods have been widely used to regulate the conformation of soybean protein in order to improve its functional properties [3]. Among them, enzymatic hydrolysis technology is shown to reduce allergenicity, improve digestibility and other nutritional attributes, and is a promising strategy for modifying the properties of soybean protein gels [4]. Among many enzyme preparations, Flavourzyme is shown to increase the conformational flexibility of soybean proteins by breaking peptide bonds and exposing functional groups involved in gel network formation under neutral conditions due to the presence of both endopeptidase and exopeptidase activities [5]. Protein hydrolysates also have a wide range of applications in the field of nutrition and dietary solutions for the elderly [6]. In addition, compared to single protein compositions, complexes with polysaccharides can provide gel products with superior textural characteristics. Carrageenan, as a natural sulfated polysaccharide, is widely used in commercial production due to its excellent gelling properties. It can be classified into three types (κ-carrageenan, ι-carrageenan, and λ-carrageenan) based on structural differences, and its gelling behavior is closely related to the number of sulfate ester groups and the presence of 3,6-dehydrated galactose units [7]. Among these, λ-carrageenan, which lacks 3,6-dehydrated galactose units and has the highest sulfate ester content (>35%), is traditionally considered to have weaker gelling ability. However, this structural feature endows it with exceptional hydrophilicity and thickening capacity, making it uniquely valuable in soft gel systems [8]. Notably, most existing studies on SPI-polysaccharide composite gels focus on κ/ι-carrageenan (which have strong self-gelling ability) or single modification strategies (e.g., only enzymatic hydrolysis or only ion addition), while the regulation of λ-carrageenan-based SPI composite gels—especially via synergistic modification—remains underexplored, leaving a gap in tailoring soft gels for specific nutritional needs.

In actual food gel production, salt ions are key regulatory factors in gelation [9]. Their mechanism of action can be traced back to the Hofmeister effect, whereby different ions influence the structure of the protein hydration layer and regulate intermolecular interactions [10]. Recent studies have mostly utilized this ion-specific phenomenon to regulate the aggregation of polymer chains, thereby achieving changes in the crystallinity and structure of polymer networks [11]. However, research and practical applications related to ions have mainly focused on Ca^2+^ and Na^+^ salts, and excessive intake may lead to health risks such as kidney stones or hypertension [12]. In contrast, potassium chloride (KCl) can replace sodium salts in the food industry and thus has a potential preventive effect on cardiovascular disease [13]. Furthermore, its cation K^+^ is located at the hydrophilic end of the Hofmeister sequence and can influence protein conformation by altering the hydration state of proteins [14]. Critically, while K^+^ has been studied for its salt-replacing role, its interaction with enzymatically modified SPI—especially in the SPI/λ-carrageenan system (previous studies have demonstrated that the addition of K^+^ does not induce gel formation in λ-carrageenan alone [15])—has not been systematically investigated. In the meantime, existing studies have primarily focused on the effects of ions on composite gels, and the exploration of the synergistic mechanism of “enzymatic modification of protein structure to regulate salting-out effects” remains insufficient, particularly in the SPI/λ-carrageenan composite system, where the structure–activity relationship between protein enzymatic modification extent and the network density and texture enhancement mediated by K^+^ salting-out effects has not yet been clearly established. This gap is particularly notable for functional food development, as precise control of gel texture and nutritional safety (e.g., low sodium) is increasingly demanded for vulnerable populations.

In-depth research into the above scientific issues will help to deepen our understanding of the interaction theory of protein/polysaccharide/ion composite systems and provide key support for the precise design of specific functional foods with customized textures. In recent years, with the aging of the global population, the percentage of people over the age of 65 is expected to increase from 9.3% in 2020 to 16% in 2050, and, in particular, the number of people over the age of 80 is expected to reach 426 million [16]. This group tends to be more susceptible to high risk of dysphagia [17], and therefore has extremely stringent requirements for food texture [18,19]. Considering texture safety and nutritional value, the development of soybean protein hydrolysate/λ-carrageenan soft gels has great potential in the field of foods for people with swallowing difficulties [12]. However, current dysphagia-friendly SPI-based gels often rely on either high polysaccharide addition (which may compromise nutritional density) or single ion regulation, which fails to balance texture and sodium reduction. This highlights an urgent need for a synergistic modification strategy that integrates enzymatic hydrolysis to optimize protein functionality and KCl salting-out to enhance gel network while reducing sodium for SPI/λ-carrageenan systems.

In this study, a novel dual-synergistic modification strategy was proposed with the aim of investigating the changes in gel properties of composite gels formed by SPI (6%, *w*/*v*) and λ-carrageenan (0.8%, *w*/*v*) pretreated with Flavourzyme (0, 10, 20, 30, 60, and 120 min, 2%, *w*/*w*) and treated by immersion in KCl (12 h, 10%, *w*/*v*) solution. Degree of hydrolysis (DH) and SDS-PAGE were used to assess the enzymatic hydrolysis of SPI; the structural features and interactions of the composite gels were resolved by surface hydrophobicity (H_0_), water holding capacity (WHC), color test, Fourier transform infrared spectroscopy (FTIR), and intermolecular force measurements; scanning electron microscopy (SEM) reveals the microstructure of the gel. In addition, the cross-linking density of the gel network was verified by X-ray diffraction (XRD) and inductively coupled plasma spectroscopy (ICP-OES). The feasibility of the application of composite gels in dysphagic foods was also comprehensively evaluated by dynamic rheological measurements combined with IDDSI framework tests. This study aims to elucidate the mechanism of enzymatic pretreatment synergizing with K^+^ salting-out effect to enhance SPI/λ-carrageenan gels, which not only advances the fundamental understanding of protein–polysaccharide–ion interactions but also provides new ideas for the development of sodium-reduced, nutrition-dense dysphagia food.

## 2. Materials and Methods

### 2.1. Materials

Skimmed soybean flakes were obtained from Yihai Jiali Grain and Oil Foodstuff Co. (Qinhuangdao, China). Flavourzyme^®^ 500 MG, featuring an enzyme activity of 500 leucine aminopeptidase units per gram (LAPU g^−1^), was sourced from Novo Co. (NovoNordisk, Bagsvaerd, Denmark). Ophthalaldehyde (OPA), 8-anilinonononitrobenzene-1-sulfonic acid (ANS) were purchased from Sigma-Aldrich Chemical Co. (St. Louis, MO, USA). Potassium chloride (purity > 99.5%) and λ-carrageenan (λ-Car) were purchased from Shanghai Yuanye Biotechnology Co. (Shanghai, China). All other chemicals and solvents used were purchased from supplier stores in China and were of analytical grade.

### 2.2. Extraction of SPI

Skimmed soybean powder was dissolved in deionized water. The pH of the resulting solution was adjusted to 8.0, followed by stirring and centrifugation. The pH of the mixture was then adjusted to 4.5, with a second centrifugation performed; the precipitate was collected and washed thoroughly. The final protein content was determined to be 92.23% ± 0.02% [20].

### 2.3. Hydrolysis of SPI

A concentration of 60 mg/mL of SPI was dissolved in deionized water and stirred for a minimum of 2 h to achieve full hydration. The SPI suspension was hydrolyzed with Flavourzyme (pH 7, 50 °C) for hydrolysis times (0, 10, 20, 30, 60 and 120 min) at an enzyme/substrate (E/S) mass ratio of 2:100 (*w*/*w*), and the hydrolysate was promptly heated in boiling water for 10 min to stop the reaction, resulting in SPI, SPH10, SPH20, SPH30, SPH60 and SPH120.

### 2.4. Characterization of Hydrolyzed SPI

#### 2.4.1. Degree of Hydrolysis (DH)

The hydrolysis of proteins was detected using the OPA method [21]. 400 μL of diluted sample was taken and mixed thoroughly with 3 mL of OPA reagent for 5 s. The absorbance was measured at 340 nm as OD_standard_ immediately after 2 min of standing (UV-2600, Shimadzu Instruments Ltd., Kyoto, Japan). The standard solution used was serine solution (0.9516 meqv/L) and the blank solution was deionized water with the same treatment as above, and the absorbance OD_sample_ of the standard and the absorbance OD_blank_ of the blank were measured. The hydrolysis degree of proteins was calculated as follows:

(1) $$Serine-NH_{2}=\frac{{OD}_{sample}-{OD}_{blank}}{{OD}_{standard}-{OD}_{blank}}\times \frac{\left(0.9516\times V\times 100\right)}{\left(X\times P\right)}$$

(2) $$h=\frac{Serine-{NH}_{2}-\beta }{\alpha }$$

(3) $$DH=\frac{h}{h_{hot}}\times 100$$

V is the dilution; X is the quality of the sample, g; P is the protein content of the sample, %; the values of α, β and h_hot_ of soybean protein are 0.970, 0.342 meqv/g and 7.8 meqv/g, respectively.

#### 2.4.2. SDS-PAGE

The method reported by Dai et al. [22] was followed. Samples at a concentration of 5 mg/mL were subjected to SDS-PAGE. Protein bands were stained with Coomassie Brilliant Blue for 1 h, destained, and then stored in deionized water overnight.

### 2.5. Preparation of λ/SPI Hydrogels and K-λ/SPI Hydrogels After Immersion

A 3 mL SPI solution at a concentration of 6% (*w*/*v*) was used, along with SPH solutions subjected to different hydrolysis time treatments (obtained in Section 2.3). λ-Car powder (0.8%, *w*/*v*) was added to the above solutions, and the mixture was thoroughly mixed before being stored in a 4 °C refrigerator overnight. This yielded complex hydrogels with different degrees of protein hydrolysis, which were designated as λ/SPI, λ/SPH10, λ/SPH20, λ/SPH30, λ/SPH60, and λ/SPH120. For subsequent treatment, each complex hydrogel was taken and immersed in a 6 mL of KCl solution where the KCl concentration is 10% (*w*/*v*). The mixture was then placed in a 4 °C refrigerator for 12 h, resulting in immersed hydrogels (designated as K-λ/SPI, K-λ/SPH10, K-λ/SPH20, K-λ/SPH30, K-λ/SPH60, and K-λ/SPH120).

### 2.6. Surface Hydrophobicity (H_0_)

ANS was mixed with the sample, and the mixture was allowed to stand for 15 min under dark conditions. Subsequently, the fluorescence intensity was recorded at an excitation wavelength of 390 nm and an emission wavelength of 470 nm, respectively, and the initial slope (H_0_) was determined via linear regression analysis [23].

### 2.7. Water Holding Capacity (WHC)

The method described by Zou et al. [24] was adopted with minor modifications. Freeze-drying was employed to determine the WHC of the hydrogels. First, hydrogel samples with dimensions of 2 × 2 × 0.5 cm^3^ were weighed (W_1_). These samples were then freeze-dried for 48 h to achieve complete water removal, after which the hydrogels were weighed again (W_2_). WHC was calculated using the following formula:

(4) $$WHC(\%)=\frac{{W_{1}-W}_{2}}{W_{1}}\times 100\%$$

### 2.8. Color Test

The color of the gel was measured using a colorimeter (CR-400, Konica Minolta Inc., Osaka, Japan) and the colors were expressed as L* (brightness), a* (red/green) and b* (yellow/blue). And the whiteness was measured according to the method of Han et al. [25] with the following formula:

(5) $$Wh\mathrm{iteness}=100-{\left[{\left(100-L^{*}\right)}^{2}+{a^{*}}^{2}+{b^{*}}^{2}\right]}^{1/2}$$

### 2.9. Rheology of Gels

The rheology of the gels was measured by a rotational rheometer (Haake Mars 60, Thermo, Waltham, MA, USA). The gel sample was cut into 0.5 × 0.5 × 0.2 cm^3^ cylinders and was placed on the stage and equilibrated at 25 °C for 120 s. First, an amplitude strain scan (γ = 0.01–100%) was performed at a constant angular frequency (1 Hz) to determine the linear viscoelastic region of the sample. The dynamic viscoelastic properties were characterized using an oscillatory frequency sweep. The angular frequency was oscillated from 0.1 to 10 rad/s, apparent viscosity was measured at 0.1–100 s^−1^ and all measurements were conducted within the linear viscoelastic region at 0.5% strain to ensure the gel could be tested without disrupting its structure [26].

### 2.10. Scanning Electron Microscopy (SEM)

The S-3400N SEM (Hitachi, Tokyo, Japan) was used to observe the microstructure. Samples were coated, gold-sprayed, and examined at an accelerating voltage of 5 kV and a magnification of 2.5 k [27].

### 2.11. Fourier Transform Infrared Spectroscopy (FTIR)

The sample powder, after being freeze-dried, was mixed with potassium bromide. FTIR spectroscopy was conducted using a Nicolet IS50 FTIR spectrometer (Thermo Nicolet Ltd., Waltham, MA, USA). Spectra were acquired over the range of 4000–400 cm^−1^, with a resolution of 4 cm^−1^ and a total of 64 scans. The secondary structure of the samples was evaluated using PeakFit Version 4.12 software (SPSS, Inc., Chicago, IL, USA) [28].

### 2.12. Determination of Intermolecular Forces

Gel intermolecular forces were determined according to the method described by [29] with some modifications. Different dissociation reagents were applied to eliminate the chemical forces. S_1_: ultrapure water; S_2_: 0.086 M Tris-0.09 M Glycine-4 mM Na_2_EDTA; S_3_: S_2_ + 0.5% SDS; S_4_: S_3_ + 8 M urea; S_5_: S_4_ + 2% β-mercaptoethanol. 2 mL of dissociation reagent was added to 1.2 g of gel. The mixture was kept in a water bath set to oscillate at 25 °C for a day and subsequently centrifuged at 6439× *g* for 10 min. Using bovine serum albumin as the standard sample, the protein content in the supernatant after Coomassie Brilliant Blue staining was quantitatively analyzed via enzyme-linked immunosorbent assay. The standard curve is:

(6) $$y=0.414x+0.7602\;(R^{2}=0.993)$$

The difference in protein solubility between S_2_ and S_1_ indicates electrostatic interactions, whereas the contrast between S_3_ and S_2_ indicates hydrophobic interactions. Similarly, the difference between S_4_ and S_3_ reflects hydrogen and hydrophobic bonding; the variation between S_5_ and S_4_ indicates disulfide bonding interactions.

Gels of the same morphology were prepared to visually assess the contribution of chemical forces in maintaining their shape. Approximately 2.5 mL of reagents (S_a_: ultrapure water; S_b_: 0.086 M Tris-0.09 M glycine-4 mM Na_2_EDTA; S_c_: 0.5% SDS; S_d_: 8 M urea and S_e_: 2% β-mercaptoethanol) were added to a vessel containing 0.5 g of gel and stirred on a mixer for 1 min. The vessel was incubated for 24 h at 25 °C in a water-bath shaker. Then, photographs were taken. The rheological measurements in Section 2.9 were also used to assess the effect of different intermolecular forces on the mechanical properties of the gels. Electrostatic interactions, hydrophobic interactions, hydrogen and hydrophobic bonds, and disulfide bonding interactions were eliminated using solutions S_b_, S_c_, S_d_, and S_e_, respectively.

### 2.13. X-Ray Diffraction (XRD)

X-ray diffraction was used to visualize the crystal structure of the sample. The data of the samples were obtained at a voltage of 40 kV and a current of 40 mA in the range of 2θ of 5° to 95°. The relative crystallinity of all samples was estimated by Origin 2024 (Origin Lab Corporation, Northampton, MA, USA).

### 2.14. Microwave Digestion

Refer to the methodology outlined by Mohamed et al. [30]. A Milestone Ethos Up microwave digestion system (Milestone, Sorisole, Italy) was employed to weigh 1 g of the gel samples, both pre- and post-soaking, into a dry and clean PTFE container. Subsequently, 6 mL of HNO_3_ was added, and the container was sealed and subjected to a two-step digestion process within the microwave digestion system. The temperature of the vessel was elevated to 200 °C over a period of 15 min and then maintained for an additional 15 min to ensure complete ablation. The resulting solution was then cooled, diluted, and stabilized with 50 mL of ultrapure water.

### 2.15. Inductively Coupled Plasma Spectroscopy (ICP-OES)

The K^+^ content of the dilutions obtained in Section 2.14 was quantified by ICP-OES Agilent 5800 (Agilent, Santa Clara, CA, USA) in three parallel runs.

### 2.16. IDDSI Test

The International Dysphagia Diet Standardization Initiative (IDDSI) outlines guidelines for adjusting food texture according to food type, as well as detailed classification criteria for edible foods [31]. To evaluate their proper classification under the IDDSI system, gel samples were cut into 1 × 1 cm squares and subjected to four tests: fork drip, spoon tilt, spoon press, and fork press. In detail, the fork drip test involved placing gel samples on a fork to analyze and record flow behavior. For the spoon tilt test, samples were placed on a spoon, which was tilted to observe sliding behavior and residual material on the utensil. The spoon and fork press tests required compressing samples with a spoon or fork to examine deformation [32].

### 2.17. Statistical Analysis

All experiments were repeated at least three times. Data were analyzed using SPSS 24.0 software (SPSS, Inc., Evanston, IL, USA). Results were expressed as means and standard errors, and data were considered statistically significant when the *p* value was <0.05.

## 3. Results and Discussion

### 3.1. Characterization of Protein Hydrolysis

#### 3.1.1. Degree of Hydrolysis (DH) Analysis

**Figure 1:**
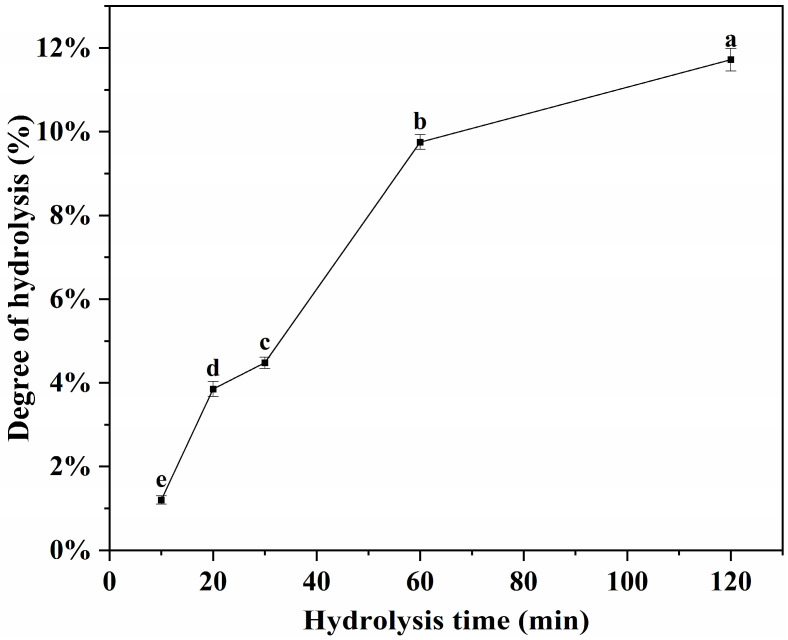
Degree of hydrolysis of SPI under different enzymatic time treatments (0, 10, 20, 30, 60 and 120 min). Different letters indicate significant differences (*p* < 0.05) between samples (a–e).

DH is a common indicator used to assess the degree of enzymatic cleavage of proteins. In general, a higher DH value indicates a higher degree of enzymatic hydrolysis, i.e., the more free amino groups are produced by enzymatic cleavage of proteins, and the generation of small-molecule peptides and free amino acids increases accordingly. As shown in Figure 1, the DH of Flavourzyme-treated soybean protein hydrolysate (SPH) increased significantly (*p* < 0.05) with the prolongation of enzyme hydrolysis time, from 1.11% at the initial (0 min) to 11.46% at 120 min of enzyme hydrolysis, and this increasing trend was in agreement with the results reported by Liu et al. [33].

#### 3.1.2. SDS-PAGE Analysis

**Figure 2:**
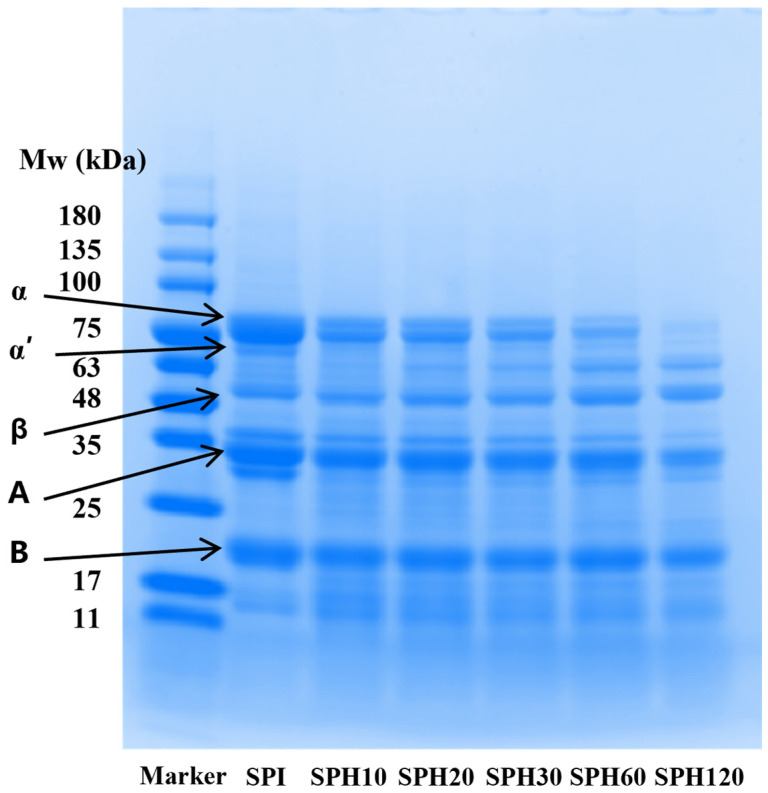
SDS-PAGE of SPI under different enzymatic time treatments (0, 10, 20, 30, 60 and 120 min).

To further investigate the effect of enzymatic treatment on SPI composition, SDS-PAGE patterns were used to characterize changes in the subunits and molecular weights of proteins under different enzymatic hydrolysis degrees. As shown in Figure 2, the protein bands corresponding to 7S (β-accompanied soybean globulin) and 11S (soybean globulin) subunits were gradually blurred and new bands appeared in the region below the molecular weights of 63 kDa and 11 kDa in SPH after hydrolysis by Flavourzyme as compared to SPI. A significant weakening of the intensity of the α and α’ subunit bands was observed with prolongation of the enzymatic hydrolysis time, marking a possible preferential action of Flavourzyme on 7S globulin. This is consistent with the study of Lv et al. [34], who showed that 7S globulin is rich in hydrophobic amino acids, and that moderate hydrolysis by Flavourzyme exposes its internal hydrophobic regions. However, under excessive hydrolysis conditions (especially SPH120 samples), 11S globin bands were also significantly degraded. In view of the fact that Li et al. [35] showed that higher 11S content contributes to the mechanical strength of the gel. Thus, the present results suggest that excessive enzymatic treatment may lead to the deterioration of gel strength. In conclusion, the degree of enzymatic hydrolysis significantly changed the subunit composition of SPI and may have an important effect on gel strength.

### 3.2. Surface Hydrophobicity (H_0_) Analysis

Enzymatic hydrolysis induces changes in protein subunits while simultaneously unfolding/exposing hydrophobic regions. Consequently, surface hydrophobicity testing is required to characterize the extent of hydrophobic region exposure. 8-Anilino-1-naphthalenesulfonic acid (ANS) specifically binds to the hydrophobic sites of proteins, and thus H_0_ is commonly used to characterize conformational changes and the degree of exposure of hydrophobic regions in protein systems [36].

**Figure 3:**
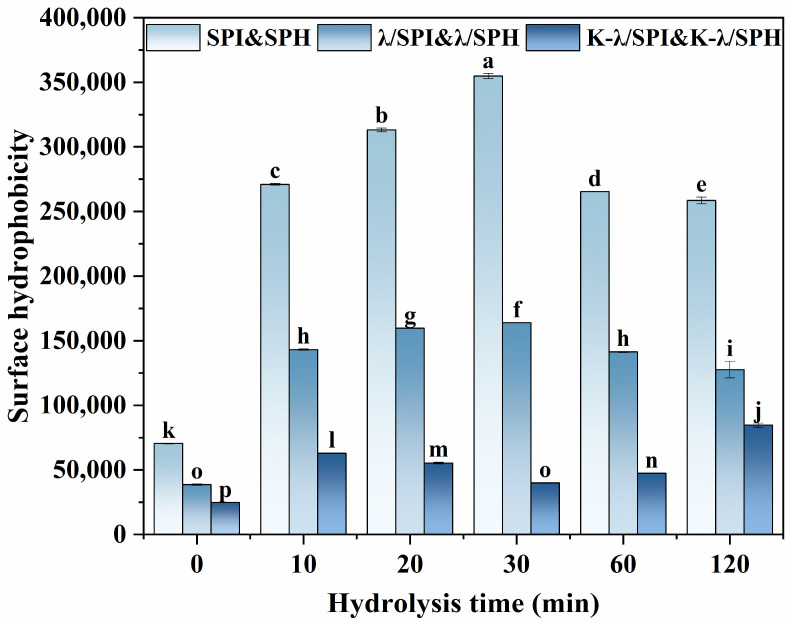
Surface hydrophobicity of SPI, λ/SPI and K-λ/SPI complexes at different degrees of enzymatic hydrolysis (0, 10, 20, 30, 60 and 120 min). Different letters indicate significant differences (*p* < 0.05) between samples (a–p).

As shown in Figure 3**,** in the SPI pure system, all SPH samples exhibited higher H_0_ compared to SPI, and mild enzymatic hydrolysis (0–30 min) caused the H_0_ value to increase rapidly first, while H_0_ showed a slow decreasing trend after excessive (60–120 min). This indicates that moderate enzymatic hydrolysis causes the protein structure to unfold, revealing more hydrophobic areas for ANS binding, thereby increasing H_0_. On the contrary, significant hydrolysis of α and α’ subunits due to excessive enzymatic hydrolysis triggers the aggregation of polypeptides via strong hydrophobic interactions, which re-conceal the ANS-binding sites, resulting in a notable reduction in H_0_ [37].

In the λ/SPI composite gel system, the H_0_ of all λ/SPI composite gels was significantly lower (*p* < 0.05) than that of SPH or SPI itself for the corresponding degree of enzymatic hydrolysis. The main reason for this was the incorporation of hydrophilic hydroxyl and sulfate groups into λ-Car, which increased the gel network’s polarity and improved the interactions between protein hydrophobic groups [12]. The result also showed that λ-Car addition led to alterations in the protein’s tertiary conformation.

The H_0_ of all samples treated with KCl solution immersion was significantly lower (*p* < 0.05) than that of their unimmersed counterparts. This indicates that the addition of K^+^ promotes the aggregation of hydrophobic regions in the system and reduces the sites available for ANS binding. In the moderate enzymatic samples (K-λ/SPI, K-λ/SPH10, K-λ/SPH10, and K-λ/SPH30), H_0_ continued to decrease after salt immersion, reaching its lowest value in the K-λ/SPH30 gel and achieving the maximum reduction in H_0_ compared with the original pure SPI/SPH systems. This may be attributed to originate from two reasons: (1) This moderate enzymatic hydrolysis exposes more hydrophobic regions and potential K^+^ binding sites, thereby enhancing hydrophobic interactions and protein–protein binding, resulting in the burial of sites available for ANS binding [38]. (2) K^+^ competes with proteins for binding water molecules, similarly to the competition between CO_3_^2-^ and SPI for water molecules as reported by Ma et al. [39] and others, which inhibits hydrophobicity contacts and interactions between amino acid residues and ANS, leading to a decrease in H_0_. In summary, the protein conformational changes induced by enzymatic pretreatment and K^+^ immersion treatment synergize to effectively regulate the H_0_ of the system.

### 3.3. Water Holding Capacity (WHC) Analysis

**Figure 4:**
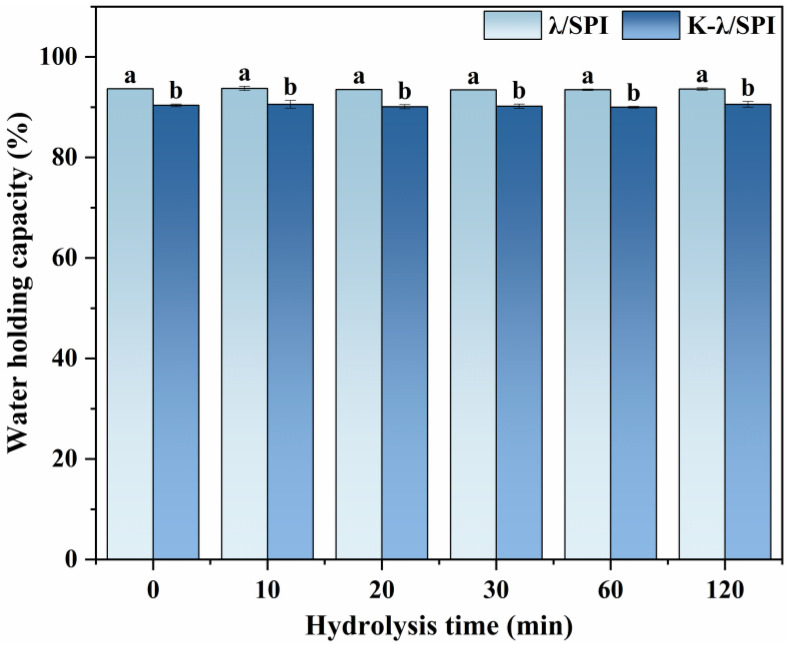
Water holding capacity of λ/SPI and K-λ/SPI gel under different enzymatic time treatments (0, 10, 20, 30, 60 and 120 min). Different letters indicate significant differences (*p* < 0.05) between samples (a, b).

WHC is important for the performance of hydrogels; the WHC of the composite gels before and after immersion treatment with KCl solution was examined. As seen in Figure 4, the WHC of the hydrogels was significantly reduced after immersion treatment with KCl solution (*p* < 0.05). This water loss is likely to originate from the K^+^-induced enhancement of polymer interchain interactions, leading to an increase in the network contraction force, which promotes water drainage [40]. Related theories suggest that water molecules as plasticizers can interact with polymer hydrophilic groups; when the water content is too high, the enhanced water-water interactions may instead weaken the polymer-polymer forces [41]. Therefore, the enhanced polymer interchain interaction, accompanied by moderate water expulsion, may be one of the important reasons for the enhancement of the mechanical characteristics of gels treated with salt, aligning with the rheological findings.

However, none of the gels treated with different enzymatic times showed a significant difference in WHC before and after KCl immersion treatment (*p* < 0.05). This may be attributed to the following reasons: (1) λ-Car, as a strongly hydrophilic polysaccharide, is the main contributor to the water-holding capacity of composite gels [42]. (2) Enzymatic treatment releases more hydrophilic groups, such as free amino groups in SPI [43], which enhances its ability to bind to water molecules. (3) Enzymatic hydrolysis weakens the contribution of SPI to gel network formation [44], which may reduce the overall WHC of the gel. The combined effect of the above can explain the phenomenon that hydrolysis treatment is not significant for WHC changes.

### 3.4. Color Analysis

Color is a key factor in evaluating the sensory quality of gels, and it can also indirectly reflect the structural density of the gel network [45]. As shown in Table 1, the Whiteness Index (WI) and Luminance value (L*) of the composite gel gradually increased with the increase in enzymatic hydrolysis time, while the red-green value (a*) gradually decreased. At 120 min of enzymatic hydrolysis (SPH120), the gel exhibited the highest L* and WI and the lowest a*. This phenomenon can be attributed to the photochemical properties of tryptophan. The side chain of tryptophan, which includes an indole aromatic ring, absorbs UV light and is liable to oxidative cleavage. This process can alter protein conformation and its UV-visible absorption spectrum, thereby affecting apparent color. Generally, higher levels of surface-exposed tryptophan tend to result in lower L* values and higher a* values [46]. Similarly, Xu et al. [47] found that the appearance of WPI gels exhibited reddening and decreased WI with the introduction of lysine. In this study, as the degree of enzymatic degradation increased (especially with SPH120), the proteins underwent significant self-assembly, resulting in the re-embedding of color-emitting amino acid residues such as tryptophan and lysine inside the aggregates, which also significantly affected the absorption and scattering behavior of the incident light. Specifically, networks that are denser or have more light-scattering interfaces usually exhibit higher L* values and WI. In summary, the results of chromatographic analyses indicate that enzymatic pretreatment can effectively modulate the exposure state of polar amino acids (e.g., tryptophan and lysine) and their aggregation behaviors in the gels and affect their optical properties by altering the microstructures of the gels.

### 3.5. Rheological Analysis

#### 3.5.1. Dynamic Rheological Analysis

**Figure 5:**
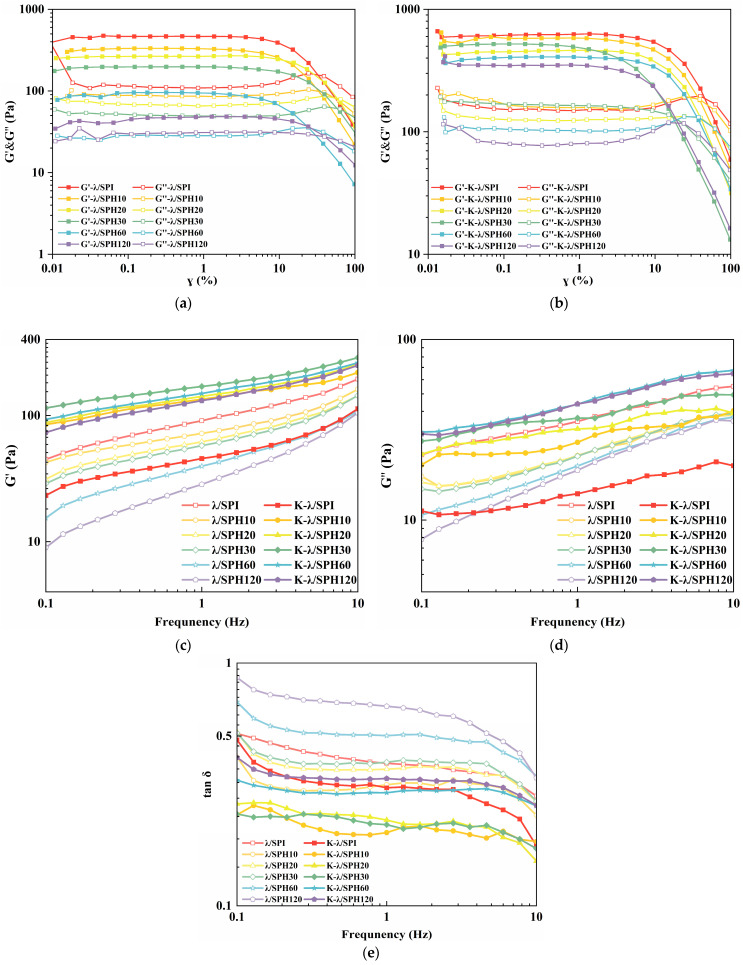
(**a**,**b**) Linear Viscoelastic Domain (LVD), (**c**) energy storage modulus (G′), (**d**) loss modulus (G″) and (**e**) the tangent of the loss angle (tan δ) of λ/SPI and K-λ/SPI gels at different enzymatic pretreatment times (0, 10, 20, 30, 60 and 120 min).

Dynamic rheological analysis was used to assess the enhancement of gel strength by enzymatic pretreatment in concert with salting-out. The energy storage modulus (G′) characterizes the solid-like elastic behavior of the gel, while the loss modulus (G″) reflects its viscous liquid behavior, and the loss tangent (tanδ), defined as the ratio of G″ to G′, is used to evaluate the relative contributions of elasticity and viscosity to the gel’s overall rheological properties. Figure 5 shows the viscoelastic properties of the gels. All the composite gels not soaked in KCl solution exhibited G′ > G″, confirming their solid-like nature. However, the tangent of the loss angle became higher (from about 0.41 to 0.87) with the prolongation of the enzymatic hydrolysis time, indicating that the gel’s solid-like elastic properties gradually diminished. This is consistent with the findings of Li et al. [48] that hydrolysis treatment of SPI acts as an inhibitor to the gel network, leading to a decrease in gel strength. After soaking in a potassium chloride solution, the gel strength is improved. At this point, the enzymatic treatment no longer weakened the gel-like solid behavior but rather enhanced its strength. The G′ values of the salt-treated gels gradually increased with the prolongation of the enzymatic hydrolysis time (K-λ/SPI, K-λ/SPH10, K-λ/SPH10, and K-λ/SPH30) and peaked at K-λ/SPH30. It is hypothesized that this may be due to the enzyme treatment leading to an expansion of the protein structure, exposing more K^+^ binding sites, which enhances the salting-out effect and strengthens hydrophobic interactions. Meanwhile, the study of Chen et al. [49] found that salt solution immersion leads to gel dehydration and promotes stronger hydrophobic chain binding, leading to increased polymer chain density and interchain friction, which in turn results in a higher G′. However, when the enzymatic hydrolysis time exceeded 30 min (K-λ/SPH60 and K-λ/SPH120), the G′ of the gels began to decrease. This may be due to excessive hydrolysis, SPH regrouped into large aggregates, which reduced the exposure of effective K^+^ binding sites and inhibited intermolecular interactions, and ultimately led to the deterioration of the three-dimensional spatial network of the gels and limited the enhancement of the gels by the salting-out effect. In conclusion, dynamic rheology suggests that enzymatic pretreatment can effectively interfere with the gel enhancement effect of the salting-out effect by modulating the protein structure.

#### 3.5.2. Apparent Viscosity Analysis

A critical element influencing the product’s texture is the gel’s apparent viscosity, which is vital for assessing dysphagic products. This study determined the apparent shear viscosity of morphologically uniform composite gels under different enzyme hydrolysis times and before and after KCl immersion [45].

**Figure 6:**
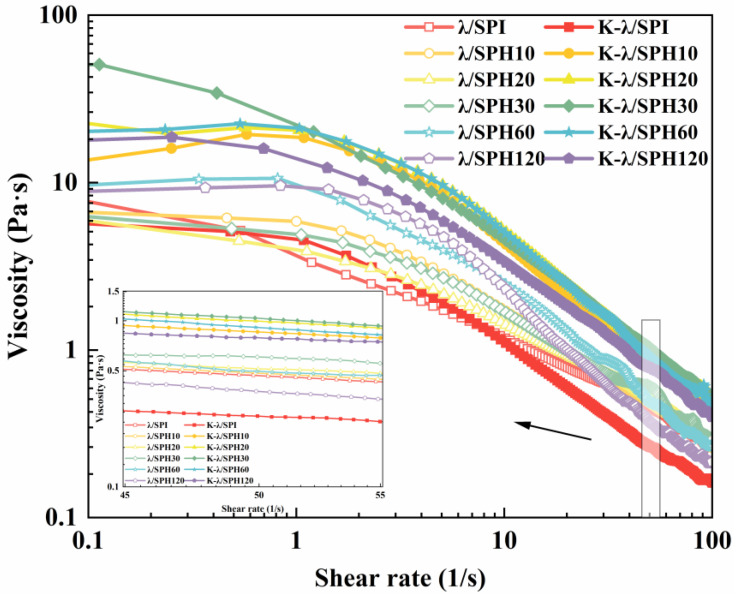
Apparent viscosity of λ/SPI and K-λ/SPI gels at different enzymatic pretreatment times (0, 10, 20, 30, 60 and 120 min). The inset shows a magnified view of the region indicated by the arrow at a shear rate of 50 1/s.

As shown in Figure 6, the apparent viscosities of all gels decreased significantly with increasing shear rate, exhibiting typical shear-thinning behavior (pseudoplastic fluid). It has been reported that push-in flow in the oral cavity occurs at a shear rate of approximately 50 s^−1^ [32]. By collecting the apparent viscosity values at 50 s^−1^ for different samples, it was noted that the gel’s viscosity initially went up and then dropped as enzymatic hydrolysis continued (0.47–0.60 Pa·s), with over-hydrolysis (>60 min) leading to the lowest value (0.38 Pa·s).

In contrast, the apparent viscosity of gels after enzymatic pretreatment combined with KCl solution immersion treatment peaked at 30 min of enzymatic hydrolysis (1.04 Pa·s) and remained high at 0.86–0.99 Pa·s for medium enzymatic (10–60 min) samples, whereas the viscosity of unenzymatic samples (K-λ/SPI) was significantly low (only 0.27 Pa·s). This demonstrated that moderate enzymatic hydrolysis treatment, synergistically with K^+^ salting-out effects, can regulate the apparent viscosity characteristics of the sample. Clinical studies have shown that increasing the apparent viscosity reduces the risk of airway infiltration and that an apparent viscosity level of 1 Pa·s significantly reduces the risk of ingested food entry into the airway, with an optimal dysphagia treatment outcome [50]. In this study, the apparent viscosity of gels treated with mild enzymatic hydrolysis combined with immersion in KCl solution matched this swallowing therapeutic threshold, predicting excellent swallowing adaptability, and was further evidenced in the subsequent IDDSI framework test (Section 3.11).

### 3.6. Scanning Electron Microscopy (SEM) Analysis

**Figure 7:**
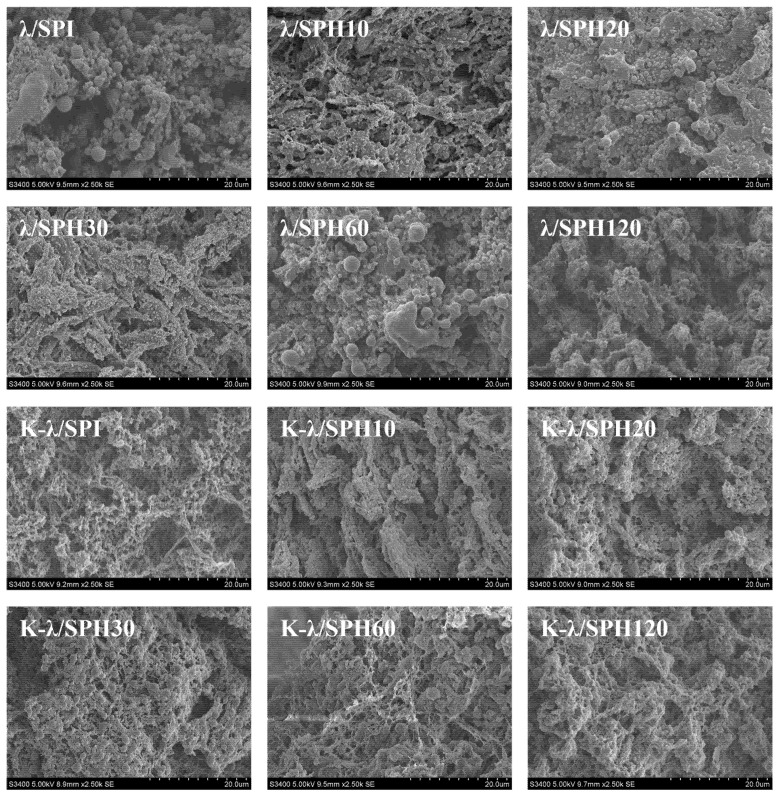
Microstructure of λ/SPI and K-λ/SPI composite gels at different enzymatic pretreatment times (0, 10, 20, 30, 60 and 120 min). SEMs at × 2.5 K.

The effect of enzymatic pretreatment combined with K^+^ immersion on the microstructure of the gels was observed using SEM. As can be seen from Figure 7, for the gels that were not treated by soaking in KCl solution, the λ/SPH gels formed under moderate enzymatic hydrolysis had a more homogeneous gel network and smaller cavities. This may be due to the fact that moderate enzymatic hydrolysis induced the structural unfolding of SPI, expanded the binding sites with λ-Car, and facilitated the cross-linking of λ/SPH gel network [51]. However, when the enzymatic hydrolysis time was extended to 60 min and above (λ/SPH60 and λ/SPH120), the gel structure gradually loosened up and became unevenly distributed. This likely stems from the excessive unfolding and subsequent reaggregation of proteins at high hydrolysis degrees, which disrupts the stabilizing intermolecular interaction forces within the system and thus reduces the homogeneity of the gel network structure [52].

For the gels after immersion in KCl, a remodeling of the original network structure with the gradual formation of a dense cross-linked structure was observed for the samples under mild to moderate enzymatic pretreatment (K-λ/SPH10, K-λ/SPH20, K-λ/SPH30, and K-λ/SPH60). This phenomenon was consistent with the results observed by Li et al. [53] in the egg yolk low-density lipoprotein/κ-Car gel system, where the gel network showed a denser structure and smaller pore sizes upon the addition of KCl solution. The progressively denser network structure is presumed to be attributed to the unfolding of the protein structure by the enzyme treatment, exposing more K^+^ binding sites, which promotes the formation of “fine-chain-like” cross-linking structures. Similarly, Liu et al. [54] reported that “thick chain” structures also appeared in the Inca peanut gel network treated with CaCl_2_ solution. In addition, Huang et al. [55] showed that the synergistic action of ionic and metal-ligand bonds could restructure and optimize the protein network. In particular, uniform and dense spatial networks were observed in K-λ/SPH30. This optimized microstructure is highly consistent with the optimal gel strength shown in dynamic rheological tests. When the hydrolysis time reaches 120 min, a homogeneous crosslinked network is no longer present and is replaced by large, irregularly distributed clusters, probably due to excessive aggregation triggered by over-hydrolysis of the proteins, which leads to the re-embedding of potential K^+^-binding sites and hydrophobic regions into the interior of the aggregates. This not only weakens key intermolecular interactions (e.g., hydrophobic interactions) but also directly leads to deterioration of gel properties.

### 3.7. Fourier Transform Infrared Spectroscopy (FTIR) Analysis

**Figure 8:**
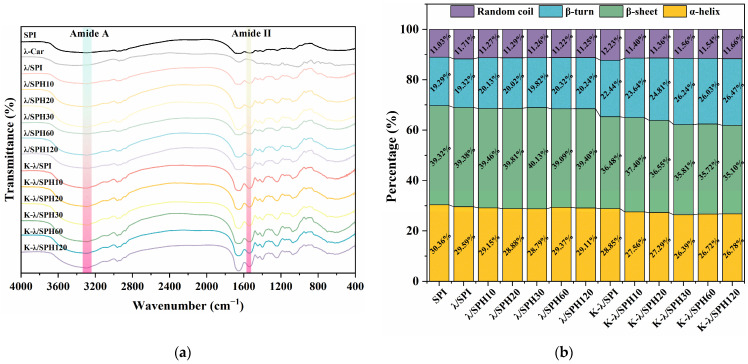
FTIR image (**a**) and second structure (**b**) of λ/SPI and K-λ/SPI composite gels at different enzymatic pretreatment times (0, 10, 20, 30, 60 and 120 min).

As shown in Figure 8a, the broad absorption peak located at 3290 cm^−1^, which is attributed to the amide A band, mainly originates from the stretching vibration of the O-H and/or N-H groups and is closely related to the inter/intramolecular hydrogen bonding network [56]. It is noteworthy that the peak intensity at 3290 cm^−1^ of the composite gel treated by immersion in KCl solution was significantly increased. This change may be related to the enhanced hydrophobic interactions between molecular chains. It is speculated that K^+^ may reduce the repulsion of surface charges on the SPI through electrostatic shielding, thereby driving protein aggregation. During aggregation, enhanced hydrophobic interactions between side chains occur alongside adjustments in the secondary structure of the main chain, leading to the formation of additional inter- and intra-chain hydrogen bonds via N-H bonds. This ultimately manifests as an increase in the peak intensity at 3290 cm^−1^ [57]. Meanwhile, in the region of the amide II band (about 1540 cm^−1^), the peak intensity increased with the prolongation of the enzymatic hydrolysis time. This likely stems from the electrostatic shielding effect of K^+^ on the SPI/λ-Car composite system, which weakens the repulsive forces between oppositely charged groups and thus enhances the intermolecular interactions. Based on the above observations, it can be inferred from this that enzymatic pretreatment in concert with K^+^ immersion treatment can affect gel strength by modulating electrostatic interactions and promoting hydrophobic interactions. However, it should not be overlooked that the variation in the peak intensity may be closely related to the quality of the up-sampled powder before each FTIR determination. So, the discussion on the interactions will be further analyzed in the intermolecular interaction forces section of Section 3.8.

Moreover, the amide I band (1600–1700 cm^−1^) underwent Fourier self-deconvolution and peak-fitting processes to quantitatively assess alterations in the protein’s secondary structure composition. As shown in Figure 8b, the content of α-helix in the samples gradually decreased, while the content of β-sheet and/or β-turn increased accordingly. This α-helix to β-structure transition is usually indicative of a certain degree of unfolding and aggregation of the protein molecule [58]. The modified conformational transition facilitates the exposure of hydrophobic groups, which promotes the formation of a more ordered and stable three-dimensional gel network structure [59]. Notably, the enhancement of gel strength could be closely associated with the increase in β-structure content. Ma et al. [39] found similar results, asserting that the enhancement in β-structure content was positively related to the strength of the gel structure.

### 3.8. Intermolecular Forces Analysis

**Figure 9:**
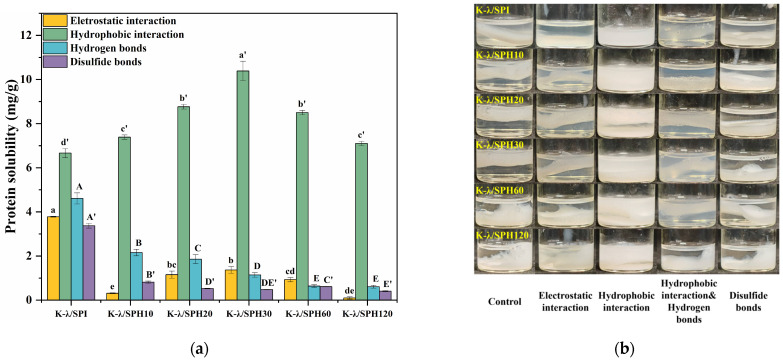

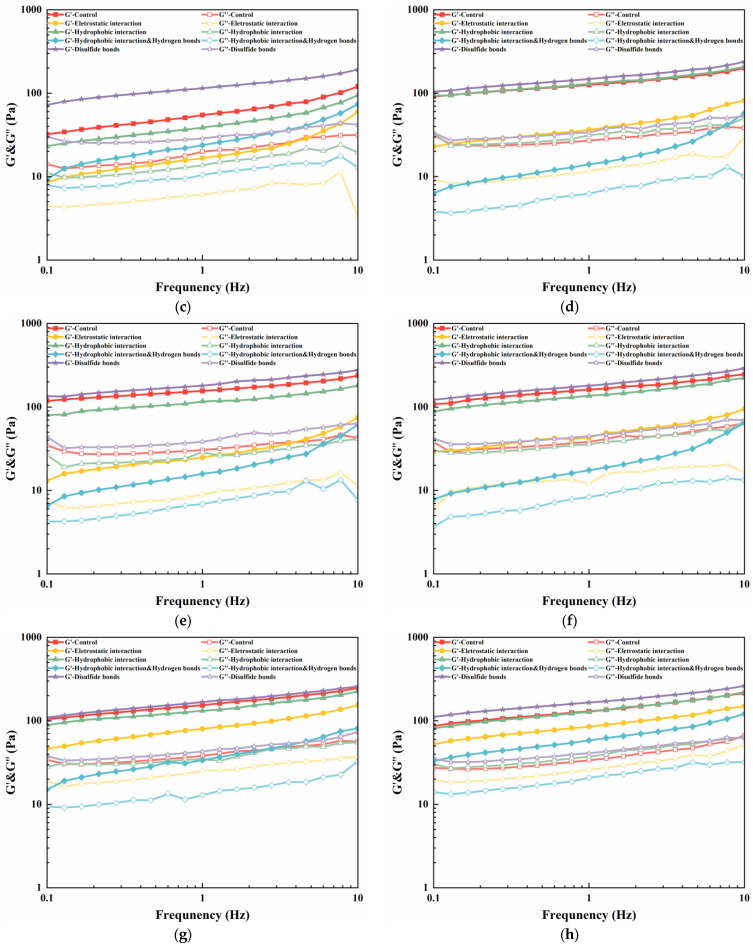
Intermolecular forces (**a**), sample image during immersion in dissociation agent (**b**) and rheological properties (**c**–**h**) of K-λ/SPI, K-λ/SPH10, K-λ/SPH20, K-λ/SPH30, K-λ/SPH60, and K-λ/SPH120 gel samples immersed in different dissociatives. Significant differences (*p* < 0.05) between samples of the same group are indicated by different letters (a–e, a’–d’, A–E or A′–E′).

Intermolecular forces in hydrogels are critical for maintaining the stability of their three-dimensional network structure. In this study, different types of interaction forces were selectively disrupted using specific dissociating agents, and the relative strength of each force was quantitatively evaluated by measuring protein solubility. The results are shown in Figure 9a, where hydrophobic interactions were identified as the main force driving protein aggregation in the K^+^ soak-treated K-λ/SPI gel system, which is critical for maintaining the overall morphological and functional stability of the gel [60]. This could be explained by the fact that salt solution immersion treatment enhances gel properties mainly by strengthening hydrophobic interactions. During the mild enzymatic stage (K-λ/SPH10, K-λ/SPH20, K-λ/SPH30, and K-λ/SPH60), the strength of hydrophobic interactions was gradually enhanced and reached a maximum at K-λ/SPH30. Subsequent over-enzymatic hydrolysis (K-λ/SPH60 and K-λ/SPH120) then led to a gradual weakening of the strength, indicating that enzymatic pretreatment was effective in the K^+^ binding efficiency and the strength of the interactions between hydrophobic groups by inducing a conformational change in the protein. This result is highly consistent with the earlier H_0_ analysis. The electrostatic interactions showed a tendency of first enhancement and then weakening with the degree of enzymatic hydrolysis, and the initial enhancement might originate from the neutralization of negatively charged amino acid residues on the protein surface by K^+^, which weakened the electrostatic repulsive force and thus promoted the intermolecular attraction [61]. Hydrogen bonding gradually weakened with increasing degree of hydrolysis, which may be related to the introduction of K^+,^ reducing the solubility of the protein, thus weakening the ability to form hydrogen bonds between polar groups. The disulfide bond contribution remained essentially stable throughout the enzymatic hydrolysis process. This was attributed to the lack of reducing activity of the Flavourzyme used to effectively break disulfide bonds. This phenomenon is similar to the ionic effect observed by Guo et al. [62] in a mung bean protein/wheat gluten composite gel system.

The intermolecular forces within a gel not only involve interactions between proteins and proteins/proteins and polysaccharides but also require consideration of interactions between polysaccharides. For a more comprehensive understanding of how internal forces within a gel influence its structure and properties, gel samples treated with K^+^ were immersed in solutions of specific dissociating agents. As shown in Figure 9b, when the gel is immersed in a dissociating agent that eliminates electrostatic interactions, hydrogen bonds, or disulfide bonds, its macrostructure remains largely intact. However, when the gel is immersed in a dissociating agent that disrupts hydrophobic interactions, its structure rapidly disintegrates, with most of the proteins dissolving into the solution. This visualization experiment further confirms that, in a gel system dominated by salting-out effects, hydrophobic interactions are the primary force maintaining protein structural stability.

At the same time, dynamic rheological measurements were performed on the gel after dissociation of specific forces, as described in Section 3.5, to assess the effects of different forces on the gel structure and rheological properties. Results in Figure 9c–h show that after selective cleavage of disulfide bonds, the gel’s G′ increased rather than decreased. This may be due to β-mercaptoethanol competing for thiol groups, thereby cleaving disulfide bonds [63]. The breaking of disulfide bonds expands the binding area, increases protein flexibility, strengthens intermolecular interactions in the system, and improves the rheological properties of the gel [64]. This also indicates that disulfide bonds do not play a major and positive role in the performance of gels. When electrostatic interactions are shielded and hydrophobic interactions and hydrogen bonds are disrupted, the rheological properties of the gel are significantly weakened. This is consistent with the slight decrease in G′ after SDS selectively disrupts hydrophobic interactions, as well as the phenomenon of a large amount of protein dissolving into the supernatant in the gel. It was possible that λ-Car strengthens the gel skeleton through hydrogen bonds and electrostatic interactions, which in turn enhances the gel network’s strength [29]. After disrupting hydrogen bonds and electrostatic interactions, the structure of λ-Car breaks down, preventing the formation of a continuous polysaccharide backbone and causing the gel network to collapse. Hydrophobic interactions are the dominant force driving protein aggregation and cross-linking with λ-Car. When hydrophobic interactions are disrupted, proteins aggregate loosely, reducing their “filling effect” within the gel network. However, since the polysaccharide backbone remains intact, this only slightly weakens the rheological properties.

Therefore, it can be hypothesized that, in the SPI/λ-Car composite gel system under salt-induced enzymatic pretreatment, the gel “scaffold” formed by λ-Car based on hydrogen bonding, electrostatic interactions, protein aggregation, and protein/polysaccharide cross-linking induced by hydrophobic interactions acts as a “filler” in the gel. The two together determine the rheological properties of the gel.

### 3.9. X-Ray Diffraction (XRD) Analysis

**Figure 10:**
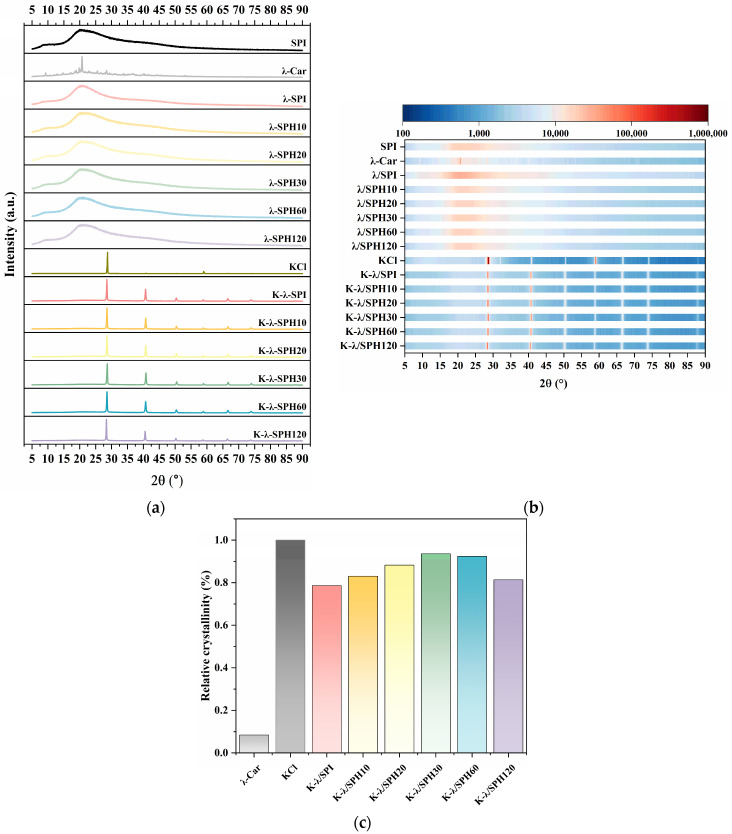
Crystal structure characterization of λ/SPI and K-λ/SPI gels at different enzymatic pretreatment times (0, 10, 20, 30, 60 and 120 min). XRD (**a**), XRD thermogram (**b**) and RC (**c**).

XRD serves to identify and assess interactions within and between molecules in polymer networks, and it determines their crystalline or amorphous characteristics [65]. As shown in Figure 10a, the XRD patterns of SPI powders showed broad diffuse peaks with no obvious diffraction peaks, indicating an amorphous structure in nature. λ-Car powders, on the other hand, showed several weak diffraction peaks, and their relative crystallinity was 8.4%, suggesting that they have a low microcrystalline structural ordering.

All the λ/SPI gels without KCl immersion showed broad peaks only at 2θ = 21°, which indicated that they basically maintained their amorphous character. For all gel samples after soaking treatment, the disappearance of the broad peak indicates that the introduction of K^+^ triggered significant molecular conformation rearrangement and ordering. Similar peaks (28.29°, 40.54°) were also observed in SPI/κ-amphibole hybrid hydrogels treated with salt solution [57]. This is attributed to the salting-out effect, which enhances interchain hydrophobic interactions, which is consistent with the results of this study. At the same time, significant characteristic peaks were observed at 2θ = 28.5°, 40.5°, 50.3°, 58.8°, 66.5°, 73.8° and 87.8°, which are close to the diffraction peak positions in the XRD spectrum of KCl, attributed to the face-centered cubic crystal lattice structure of KCl [66].

The specific details of the diffraction peaks can be visualized by the XRD band heat map in Figure 10b. The main diffraction peak located at 2θ = 28.54° was gradually shifted to a higher angle and reached a maximum value (28.65°) at K-λ/SPI30 in the moderate enzymatic samples (10–30 min), whereas the excessively digested samples (60–120 min) resulted in the peak being shifted to a lower angle. According to Bragg’s equation (nλ=2dsinθ), an increase in the diffraction angle θ implies a decrease in the crystal plane spacing. Therefore, the crystallite spacing showed a trend of decreasing and then increasing with the prolongation of the enzymatic hydrolysis time, reflecting the corresponding change in the average distance between molecular chains. The trend of decreasing and then increasing the crystallite spacing with the increase in hydrolysis time similarly indicates the change in intermolecular distance in the same trend.

Given that the unsalted gels were all amorphous, only the KCl-soaked treated samples were analyzed for relative crystallinity. As shown in Figure 10c, the crystallinity trend was similar to the microstructural evolution observed by SEM. At moderate enzymatic hydrolysis (10–30 min), the crystallinity gradually increased and peaked at K-λ/SPH30 (93.57%), while it decreased to 81.34% after excessive enzymatic hydrolysis (60–120 min). This may be due to the fact that moderate enzymatic hydrolysis exposed more K^+^ binding sites on the protein surface, enhancing the ion-induced salting-out effect. This intensified the polymer inter/intra-chain interactions (e.g., hydrophobic interaction, electrostatic attraction) and promoted the formation of a more ordered and denser lattice. This dense structure may be the microscopic basis for the highest gel strength of K-λ/SPH30 [33]. This finding echoes the conclusion of Lu et al. [67] that the salting-out effect of sodium citrate (Na_3_Cit) not only enhances the crystallinity of the PVA/starch system, but also strengthens the inter-component interactions through hydrophobic interaction. Excessive protein aggregation due to over-enzymatic hydrolysis may mask the effective K^+^ binding sites and impede the ordered arrangement of the polymer chains, thus weakening the salting-out effect and decreasing the crystallinity, which is consistent with the large-sized irregular aggregates observed by SEM. It is worth noting that, although the literature reports that high concentrations of Na^+^ can form new amorphous structures with low crystallinity through intermolecular interactions [68]. Considering that SPI itself is amorphous and λ-Car crystallinity is low, it is usually difficult to achieve such high crystallinity (e.g., 93.57%) for protein/polysaccharide complexes by conventional salt precipitation strategies. It is possible that the gel adsorbed and encapsulated some KCl crystals [69]. Therefore, the significant difference in crystallinity may originate from the difference in the actual K^+^ binding concentration/efficiency due to enzymatic pretreatment, to be further verified by subsequent elemental analysis (Section 3.10).

In summary, XRD analysis indicated that enzymatic pretreatment could effectively regulate the crystal morphology and degree of crystallinity of the K^+^-induced λ/SPI composite system and affect its functional properties by reconfiguring the gel network.

### 3.10. Elemental Analysis

To quantitatively assess the salting-out effect and to investigate the distribution of K^+^ in the gel network, inductively coupled plasma emission spectroscopy (ICP-OES) was used to determine the K^+^ content in the gels after KCl immersion treatment. ICP-OES was chosen based on its good accuracy and suitability for the analysis of higher concentration samples (compared to ultra-trace ICP-MS [70]). The samples were pretreated by microwave hydrolysis to completely decompose them since the SPI preparation process may introduce background Cl^−^, interfering with the analysis, even though a consistent relative concentration in the system would not bring about a significant difference in Cl^−^ concentration. Therefore, the K^+^ content is a more direct indicator to characterize the salt ion binding and salting-out modulation.

**Figure 11:**
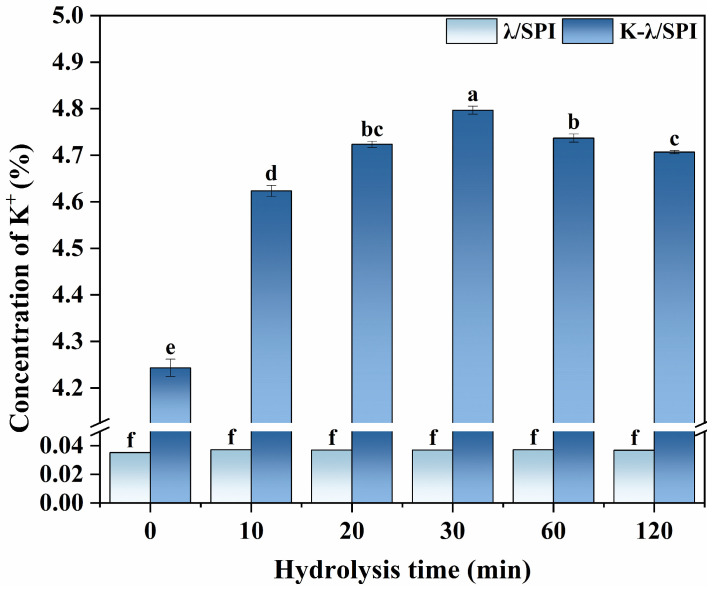
Concentration of K^+^ in λ/SPI and K-λ/SPI gels at different enzymatic pretreatment times (0, 10, 20, 30, 60 and 120 min). Different letters indicate significant differences (*p* < 0.05) between samples (a–f).

As shown in Figure 11, the K^+^ content of all samples not treated with KCl solution immersion was 0.03%, which was not significantly different, indicating that different enzymatic conditions combined with KCl solution immersion do not lead to additional K^+^ introduction. When treated with salt solution immersion, the K^+^ concentration was found to be significantly higher (*p* < 0.05) at the stage of moderate enzymatic hydrolysis (10–30 min), elevating from 4.2% for K-λ/SPI to 4.80% for K-λ/SPH30. When over-enzymatic treatment (60–120 min) was applied, the K^+^ concentration significantly decreased (*p* < 0.05) to 4.7% of K-λ/SPH120. This is consistent with the XRD crystallinity results that moderate enzymatic hydrolysis exposes more protein binding sites, enhances the K^+^ binding capacity, and increases the amount of K^+^ in the gel system. The reaggregation of proteins after over-enzymatic hydrolysis hindered K^+^ binding. The presence of K^+^ could neutralize the negative surface charges of λ-Car and SPI through electrostatic shielding, weakening electrostatic repulsion to promote molecular proximity and cross-linking, which could contribute to the interaction between SPI and λ-Car. Similarly, Zhang et al. [71] found that a dense cross-linked network inhibits Ca^2+^ diffusion from the immersion solution into the gel interior.

The changes in K^+^ content determined by ICP-OES indicated that enzyme pretreatment can effectively regulate the binding efficiency and retention rate of K^+^ in the gel by adjusting the gel network structure, thereby mediating the evolution of its microstructure to produce a “steric hindrance” effect and enhance its macro performance [72].

### 3.11. IDDSI Analysis

The IDDSI framework provides global standards and test methods for the assessment of dysphagic foods. In this study, fork pressure test, fork drop test and spoon tilt test were conducted to assess the suitability of SPI/λ-Car composite gel as a dysphagic food [31].

**Figure 12:**
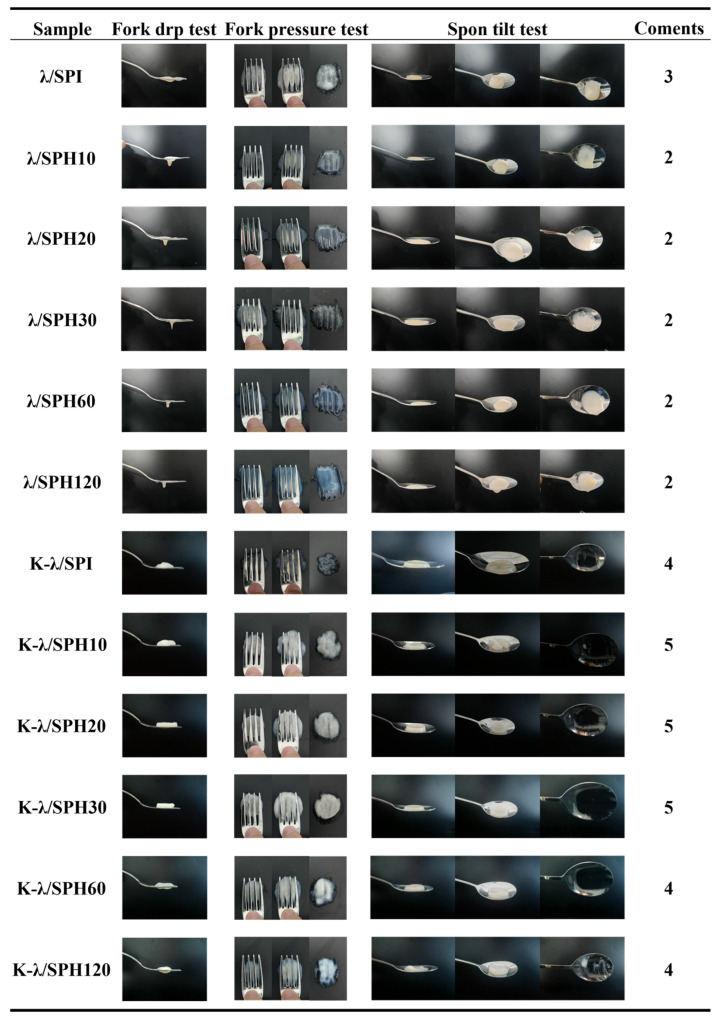
IDDSI test of λ/SPI and K-λ/SPI gels at different enzymatic pretreatment times (0, 10, 20, 30, 60 and 120 min).

The results are shown in Figure 12, for all enzymatic samples not treated with KCl immersion in the fork-drop test easily dripped from the fork gap, exhibiting excessive fluidity, which is closely related to the weak solid behavior in the rheological test. According to the IDDSI framework, such gels were categorized as Level 3 (liquid) foods. The unenzymatized and unsoaked gel, although it behaved as a Level 3 or higher food in the fork drop test, left a visible trace of gel on the spoon surface during the final stage of the spoon tilt test, indicating excessive viscosity. This high viscosity requires greater tongue muscle thrust to push the food into the pharynx during swallowing and may result in sticking to the tongue and pharynx during pushing the food to the pharynx, increasing the risk of aspiration or choking, making it unsuitable for use as a dysphagia product.

For all gels treated by immersion in KCl solution, they passed the fork-drop test and the spoon tilt test and performed at Level 4 (slushy/very thick) food or better. Under the fork-press test, all samples passed through the fork gap and were all easily mashed with a fork, causing no whitening of the thumbnail bed in the process and forming a clear indentation pattern on the sample surface, indicating that their particle sizes were all within safe limits [73]. It is noteworthy that the samples under moderate enzymatic treatment, especially K-λ/SPH30, had the least amount of food fragments after fork compression and the indentation was able to recover quickly, suggesting that they have the best cohesive properties, which is in line with the requirements of a level 5 (finely crumbled/wet and soft) dysphagia diet. This good cohesion is effective in avoiding the risk of physical obstruction due to fragmentation of food during swallowing [32].

### 3.12. Mechanisms Proposed

**Figure 13:**
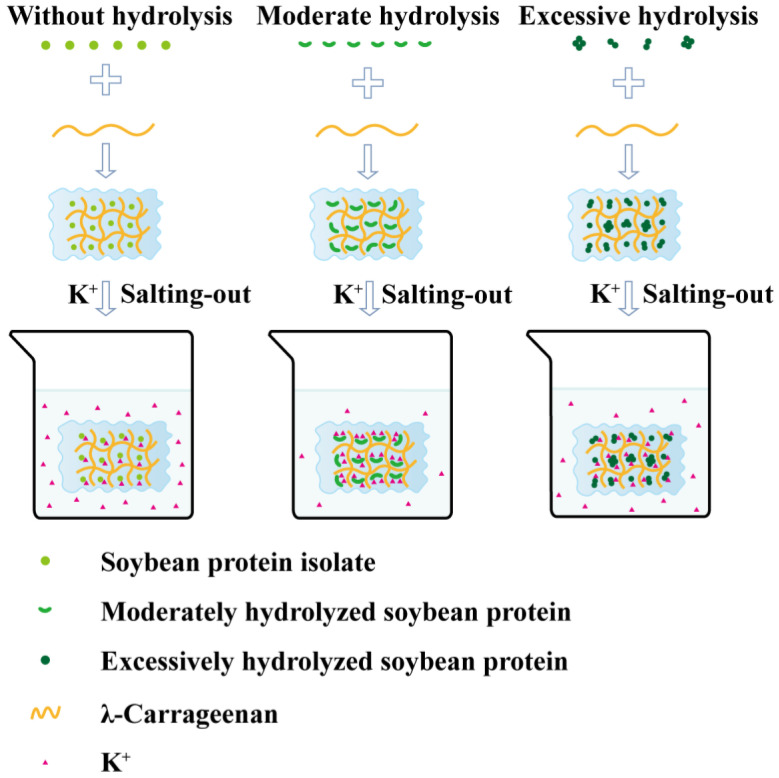
Mechanism of enzymatic pretreatment synergizing K^+^ immersion to enhance gel properties.

The mechanism underlying the formation and enhancement of SPI/λ-Car composite gels is depicted in Figure 13. As a strongly hydrated cation in the Hofmeister series, K^+^ significantly facilitates protein gelation by shielding electrostatic repulsion within the gel and, simultaneously, enhancing hydrophobic interactions. Hydrolysis pretreatment with Flavourzyme alters the morphological characteristics of proteins. Moderate hydrolysis promotes protein molecule unfolding, exposing more concealed hydrophobic regions and potential K^+^ binding sites; in contrast, excessive hydrolysis triggers excessive protein aggregation, which reburies hydrophobic groups and K^+^ binding sites, thereby modulating K^+^ binding efficiency. For samples moderately hydrolyzed by Flavourzyme, K^+^ addition more effectively induces the formation of numerous ordered protein aggregates via hydrophobic cross-linking. These aggregates, serving as “fillers” in the gel network, work synergistically with the λ-Car network “skeleton” (maintained through hydrogen bonds and electrostatic interactions) to collectively form a densely cross-linked gel network. The strengthened “steric hindrance” effect further supports high-concentration K^+^ retention in the network structure. Under conditions of improved K^+^ binding/retention, the salting-out effect is enhanced, with both crystallinity and interaction strength increased—ultimately achieving optimal gel performance and swallowing adaptability (IDDSI Level 5).

## 4. Conclusions

In this study, we investigated the effect of enzymatic pretreatment of soybean protein isolate (SPI) synergized with K^+^ salting-out on the structure and properties of composite gels. First, moderate enzymatic treatment optimized SPI’s spatial conformation, leading to partial decomposition of the 7S subunit and increased surface hydrophobicity. Second, the immersion treatment formed a dense cross-linked gel structure with a protein network “filler” induced by hydrophobic interactions and a λ-carrageenan gel “skeleton” maintained by electrostatic interactions and hydrogen bonding, as well as producing more β-regions. Enhanced salting-out effect promoted higher crystallinity and K^+^ content. Ultimately, this study demonstrated that enzymatic hydrolysis pretreatment combined with salting-out improved the gels’ rheological properties and achieve better swallowing suitability. In the future, the synergistic effect of different Hofmeister ions with the enzymatic pretreatment of proteins can be investigated, thus providing further theoretical and practical references for the design of innovative food products for dysphagia patients.

## Figures and Tables

**Table 1:** Color of λ/SPI and K-λ/SPI gels at different enzymatic pretreatment times (0, 10, 20, 30, 60 and 120 min).

GEL	L*	a*	b*	WI
λ/SPI	29.07 ± 0.49 ^g^	−0.3 ± 0.056 ^a′^	2.53 ± 0.533 ^C^	29.02 ± 0.493 ^G′^
λ/SPH10	31.23 ± 1.714 ^f^	−0.82 ± 0.06 ^b′c′d′^	2.91 ± 0.106 ^B^	31.17 ± 1.71 ^F′^
λ/SPH20	30.44 ± 0.638 ^f^	−0.79 ± 0.04 ^b′c′^	2.16 ± 0.132 ^D^	30.4 ± 0.642 ^F′^
λ/SPH30	30.59 ± 0.964 ^f^	−0.21 ± 0.537 ^a′^	5.37 ± 0.122 ^A^	30.38 ± 0.957 ^F′^
λ/SPH60	34.89 ± 0.182 ^d^	−0.97 ± 0.006 ^c′d′^	1.30 ± 0.076 ^F^	34.87 ± 0.181 ^D′^
λ/SPH120	40.67 ± 1.247 ^a^	−1.98 ± 0.015 ^g′^	−0.73 ± 0.102 ^H^	40.63 ± 1.245 ^A′^
K-λ/SPI	33.57 ± 0.346 ^e^	−0.62 ± 0.015 ^b′^	1.71 ± 0.087 ^E^	33.55 ± 0.344 ^E′^
K-λ/SPH10	35.04 ± 0.346 ^d^	−1.24 ± 0.012 ^e′f′^	−0.56 ± 0.118 ^H^	35.02 ± 0.346 ^D′^
K-λ/SPH20	34.98 ± 0.410 ^d^	−1.38 ± 0.026 ^f′^	−1.25 ± 0.060 ^IJ^	34.95 ± 0.409 ^D′^
K-λ/SPH30	38.02 ± 0.455 ^c^	−1.05 ± 0.055 ^d′e′^	0.78 ± 0.078 ^G^	38.01 ± 0.457 ^D′^
K-λ/SPH60	35.84 ± 0.201 ^d^	−1.41 ± 0.035 ^f′^	−1.16 ± 0.049 ^I^	35.82 ± 0.200 ^D′^
K-λ/SPH120	39.40 ± 0.682 ^b^	−2.07 ± 0.067 ^g′^	−1.49 ± 0.121 ^J^	39.35 ± 0.682 ^B′^

Significant differences (*p* < 0.05) between samples of the same group are indicated by different letters (a–g, a′–g′, A–J or A′–F′).

## Data Availability

The original contributions presented in this study are included in the article. Further inquiries can be directed to the corresponding authors.
